# Machinability of Minor Wooden Species before and after Modification with Thermo-Vacuum Technology

**DOI:** 10.3390/ma10020121

**Published:** 2017-01-28

**Authors:** Jakub Sandak, Giacomo Goli, Paola Cetera, Anna Sandak, Alberto Cavalli, Luigi Todaro

**Affiliations:** 1 CNR IVALSA, San Michele all’Adige (TN) 38010, Italy; sandak@ivalsa.cnr.it (J.S.); anna.sandak@ivalsa.cnr.it (A.S.); 2 Faculty of Mathematics, Natural Sciences and Information Technology, University of Primorska, Glagoljaška 8, 6000 Koper, Slovenia; 3 GESAAF University of Florence, Firenze 50145, Italy; albertocavalli75@gmail.com; 4 SAFE University of Basilicata, Potenza 58100, Italy; paola.cetera@unibas.it (P.C.); luigi.todaro@unibas.it (L.T.)

**Keywords:** machinability, wood, hygro-thermal modification, poplar, cedar, pine, alder, roughness, surface quality

## Abstract

The influence of the thermal modification process on wood machinability was investigated with four minor species of low economic importance. A set of representative experimental samples was machined to the form of disks with sharp and dull tools. The resulting surface quality was visually evaluated by a team of experts according to the American standard procedure ASTM D-1666-87. The objective quantification of the surface quality was also done by means of a three dimensions (3D) surface scanner for the whole range of grain orientations. Visual assessment and 3D surface analysis showed a good agreement in terms of conclusions. The best quality of the wood surface was obtained when machining thermally modified samples. The positive effect of the material modification was apparent when cutting deodar cedar, black pine and black poplar in unfavorable conditions (i.e., against the grain). The difference was much smaller for an easy-machinability specie such as Italian alder. The use of dull tools resulted in the worst surface quality. Thermal modification has shown a very positive effect when machining with dull tools, leading to a relevant increment of the final surface smoothness.

## 1. Introduction

Wood as a natural resource possesses several advantages as a sustainable material for construction and aesthetical appeal. Unfortunately, wood is considered as a relatively low-durability material and requires special care to assure a long-term service life. For that reason, improvements of selected wood properties by diverse modification procedures have recently become a common trend [1,2]. High-value wood resources might therefore be obtained with different processes, including impregnation, chemical modification or thermal treatments, among others. The potential economic gain is more attractive when considering less-often-used or minor wood species of relatively low market value. Thermo Hydrous modification of wood (TH) is an example of a successful implementation of modification technologies, with volumes of thermally treated timber gradually increasing worldwide. Thermal treatment as a means to enhance the properties of lesser-used species has already been used. A trial for the enhancement of Turkey oak (*Quercus cerris* L.) through vacuum thermal modification was shown to improve the characteristics of the material [3,4]. On the other hand, extensive changes to the native wood properties, such as improvement of the durability, reduced shrinkage/swelling and a more attractive appearence, result in a general modification of the mechanical properties. In this framework, an experimental trial to study the effect of thermal modification on the machinability and final quality after machining of some local species was conducted. In the field of TH of wood, not much information can be found as related to the analysis of its cutting. Some general rules regarding handling and machining of ThermoWood^®^ were provided in the form of a technical manual by [5]. It included general recommendations considered important in order to assure the proper handling of basic machining operations such as planing, milling or sanding in order to minimize damage of the wood due to processing. Studies on the surface quality after machining thermally modified *Eucalyptus grandis* and *Pinus caribaea* var. hondurensis were conducted by [6]. The final quality, assessed visually after planing thermally treated timber, was higher compared to the control samples. However, the surface roughness after sanding was smoother on control samples than on thermally modified samples (average roughness *Ra* = 7 and 12 μm, respectively). 

The opposite results were reported by [7], even if the very short measurement length of the surface profiles (*l* = 0.25 mm) makes the results questionable, especially when referring the surface roughness parameters to human visual perception. Higher numbers of excellent-surface-quality specimens after machining thermally modified wood, compared to controls for planing, sanding, boring and shaping, were recorded following visual assessment by [8]. It was also concluded that mortising was not noticeably influenced by the thermal modification process, while the surfaces of untreated samples after turning were considered smoother than of those of thermally modified samples. Further tests for surface quality assessment after machining of thermally modified *Eucalyptus grandis* were executed by [9,10]. Nevertheless, in both studies, the treated material was not compared to an untreated control.

It can therefore be assumed that machining of thermally modified wood results in a superior surface smoothness when compared to untreated wood, with the exception of turning operations. However, there is a lack of systematic studies allowing a broad understanding of the thermal modification effects on the machinability of wood. No studies on the grain orientation’s (intended as the general direction the cells composing the wood are oriented) influence on the presence of surface defects have been reported so far for these novel materials. Consequently, the goal of this work was to objectively quantify the influence of the thermal treatment on the machined surface smoothness considering the grain orientation as an important factor. The other objective was to compare the numerical analysis of roughness with the expert’s panel assessment. Besides visual analysis, the method proposed by [11] was used. The method was already successfully applied to evaluate the machinability of Douglas fir and Aleppo pine [12].

## 2. Experimental Section

### 2.1. Thermal Treatment of Experimental Samples

Wooden boards of 35 × 200 × 2500 mm^3^ (thickness × width × length, respectively) made of deodar cedar (*Cedrus deodara* Roxb.), black pine (*Pinus nigra* Arnold.), black poplar (*Populus nigra* L.) and Italian alder (*Alnus cordata* Loisel) were used as experimental samples. The timber originated from Southern Italy forests located in the Basilicata Region. All the species investigated, even if available in relatively high quantities, are considered so far as minor and of low economic importance. This research project was dedicated, beside of technical characterization of thermally treated wood machinability, to the development of new application fields for that resources.

**Figure 1:**
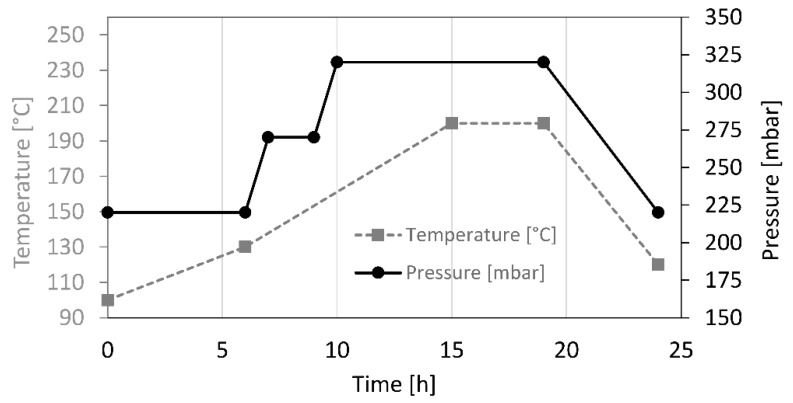
Chamber temperature and pressure during the Thermo-Vacuum (TermoVuoto) treatment of the samples.

Each wooden board was spitted in two parts in order to obtain twin samples, corresponding to control (un-treated) and modified sample sets. The modification was achieved by thermal modification using a Thermo-Vacuum (Termo Vuoto) plant produced by WDE Maspell srl. (Terni, Italy). The modification was conducted by ALAC srl (Recanati, Italy). Each species was processed in different batches, nevertheless the treatment process parameters were identical each time. Both, wood drying and effective thermal modification were performed in the same processor (autoclave). The process was performed at variable pressure and temperature, according to the schedule presented in Figure 1. Wood was firstly dried for 4 h in vacuum conditions (185–200 mbar) at a temperature of 90 °C. The thermal modification stared after drying by gradually increase the temperature till 200 °C in 15 h. The 200 °C were then held for four additional hours. Finally the timber was cooled to ~100 °C in ~5 h. More details regarding the Thermo-Vacuum process and its technical particularities are described in the literature by [13,14]. The wood density (ρ) was measured before and after modification at environmental standard conditions (20 °C if temperature (T) and 65% of relative humidity (RH)), see Table 1. The samples were conserved inside a conditioned room at 20 °C and 65% RH and moved with envelops to the workshop for machining. The moisture content of the samples was determined, just before the machining process, by gravimetric method according to the European standard EN 13183-1:2003. Four samples for each condition were used and the samples were extracted from the remains after machining. The moisture content values are reported in Table 2.

### 2.2. Wood Machining Test

The procedure for processing and characterize the samples was the same as described by [11]. Tangential boards were chosen for machining and a set of wooden disks was cut from the experimental material on a Computer Numerical Controlled (CNC) routing machine. The diameter of each disk was *D* = 130 mm, what corresponds to a circumference length of ~400 mm. Four disks were machined from each wooden board in sequence. The processed board was tightly fixed to the machine table with screws in order to avoid undesired movement of the disk during machining. Support layer of fiberboard panel was applied to allow the tool to cut the whole board thickness. The cutting tool was a high speed steel router bit with 14 mm of diameter, three helical cutting edges and an a positive helical angle (λs') of 20°. The cutting configuration was up-milling, the feed speed *Vf* = 8 m·min^−1^ and the cutting speed *Vc* = 13.2 m·s^−1^ corresponding to 18,000 RPM of the spindle. The tool used for experimental cutting was previously engaged by industrial partner for machining Medium Density Fiberboard (MDF) of the constant thickness (18 mm). Consequently, the initial part of the cutting edge become dull as a consequence of the excessive usage. The remaining portion of the cutting edge, not involved in the MDF machining, was therefore intact and corresponded to the freshly sharpened tool (radius of the cutting edge ~5 μm). The extent of the wearing of tip profile was not directly measured but a clear polishing of the cutting edge zone, typical for excessively used tools, was visually noticed in the dull part of the tool. Such configuration allowed to obtain more defects on the dull part as well as to check the effect of the cutting edge sharpness as well. The comparison between different species was done with the dull tool in order to magnify the defects. A comparison between sharp and dull tool was made on pine because presenting the higher difference in terms of final quality between the parts machined with dull and sharp tools compared to the other species.

Special care has been paid to select boards with four to eight early rings appearing on the cross section. Thirty-two disks (4 species × 2 treatments × 4 replicates) were machined. All samples were conditioned before and after machining at air temperature 20 °C and 65% of relative humidity.

### 2.3. Visual Assessment of the Surface

**Figure 2:**
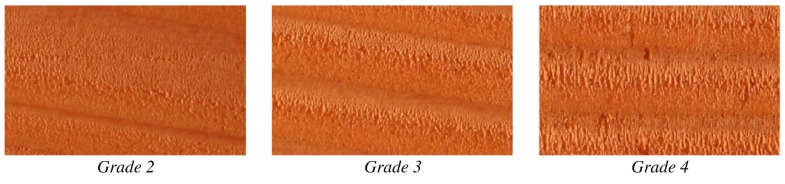
Classification of “pressed grain”, a defect not previously classified by ASTM D-1666 87 (2×).

The visual assessment of the machined surface was done after reconditioning of the samples at 20 °C and 65% RH and conducted according to the ASTM D-1666 87 [15]. The standard defines four main groups of defects, including raised grain, fuzzy grain, torn grain and chip marks. The standard defines the defects as follows: raised grain—a roughened condition of the surface of lumber in which the hard summerwood is raised above the soft springwood but not torn loose from it; fuzzy grain—small particles or groups of fibers that did not sever clearly in machining but stand up above the general level of the surface; torn grain—that part of the wood torn out in dressing; chip marks—shallow dents in the surface caused by shavings that have clung to the knives instead of passing off in the exhaust as intended. Occurrence and magnitude of the defects must be evaluated with the aid of photographic references and classified in five grades. At the extremes grade 5 corresponded to highly defected surface, where grade 1 was assigned to the defect free surface. Presence of chip marks was ignored in this study as it appears mainly due to particular cutting configuration and/or tool characteristics not relevant in this research. Because the defects classified by the ASTM standard does not cover all the possible defects two additional defects were classified: “pressed grain” and “tilted grain”. Pressed grain was evaluated according to Figure 2, tilted grain, whom a reference is reported in [16], was not observed.

The effect of grain orientation (φ) was considered by evaluating the surface smoothness independently on eight sectors of 45° over the disk circumference. Being the grain orientation going from 0° to 180° the same as gong from 180° to 360° on one disk there is the replica of two experiments.

The visual analysis was conducted by a group of trained experts on a single disk at once, considering every sector separately and repeating the assessment independently on each replica. The results of evaluation were recorded on the report and further statistically processed. The final rating of the surface quality was computed by averaging the values of every sector and by summing the resulting four values (one for sector). The “no defect” case would result in a score of 4 (grade 1—on four sections). On the other hand, the worse defect appearing on all disks would result in a score of 20 (grade 5 perceived on four sectors). The lower is the rating the higher the surface quality, with a scores ranging from 4 to 20.

### 2.4. Surface Topography Measurement and Analysis

Visual assessment of wood surface quality, even if performed by properly trained evaluators, is a fairly subjective routine. Thus, an objective evaluation of the same samples by means of dedicated instrumentation, followed by a 3D surface reconstruction and mathematical analysis was performed as an alternative to human inspection. The sample disks were reconditioned to 20 °C and 65% RH, drilled in their geometrical centre and installed on the rotary stage of a custom roughness scanner [11]. The 3D surface geometry was extracted by applying a triangulation algorithm based on the laser line projected on the surface. The line section was illuminated by means of 1 mW laser projector Lasiris (Stocker Yale, Salem, New Hampshire , USA), emitting visible light λ = 635 nm in a form of ultra-thin (~10 μm width) line. The image of the laser light section was acquired by a high resolution CCD camera (Pixelink PL-A782, Ottawa, Ontario, Canada) equipped with a zero distortion multi-configuration macro lens MC3-03X (Optoengineering, Mantova, Italy). The incidence angle of illumination was 45°, while the reflectance angle was 0°. The disk was rotated with 0.5° increment and the image of the laser line projected on the disk side was acquired each time. The surface profile was computed by applying the centre of gravity algorithm [17]. The resulting 3D surface map was interpolated into a matrix of 3000 × 720 pixels, corresponding to 720 profile lines along the disk circumference composed 3000 points each. Considering that the scanning width of camera was 16.7 mm, the spatial resolution of the profiles was 5.6 µm.

The rough surface map was pre-processed by filtering in order to isolate the roughness signal related to the surface quality. A wavelet transform was implemented here with the wavelet type DB03 applied at the level 4.0 to remove the error of form. Secondly, high frequency noise was removed after implementing wavelet type DB02 at a level 4.0. Using wavelets for filtering is normalized and defined by ISO 16610-29 for profiles, and for surfaces it will be defined by the ISO 16610-69 currently under development. The pre-processed surface was finally divided in single profiles for different grain orientations and on each profile the roughness level was quantified by means of the *Ra* computer according to Equation (1):

(1) $$Ra=\frac{1}{n}\sum_{i=1}^{n}\;\left|y_{i}\right|$$

where *n*—number of points in the profile, *y_i_*—distance between surface profile point and the mean line.

## 3. Results and Discussion

### 3.1. Visual Assessment

**Figure 3:**
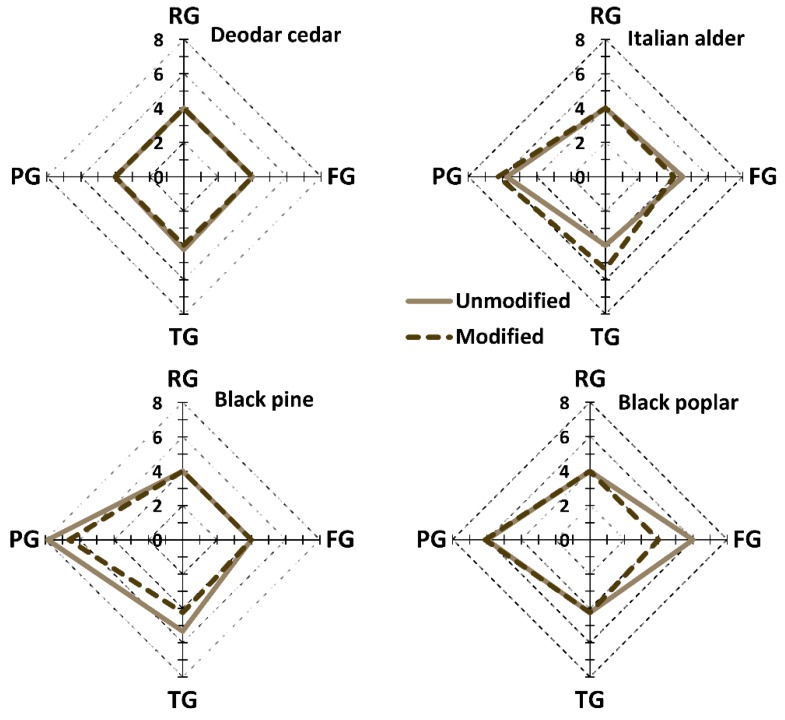
Results of visual quality assessment of wooden disks’ surfaces considering: raised (RG), fuzzy (FG), torn (TG) and pressed (PG) grains. Note: the lower the value, the smoother the surface is; a value of 4 indicates that the surface is defect-free.

The results of the visual assessment are summarized in Figure 3 in the form of radar charts. The extent of the four types of surface defects is presented, including raised, fuzzy, torn and pressed grains.

The analysis of the deodar cedar highlighted a slight tendency to form torn grains when processing control samples. On the other hand, TH cedar resulted in a perfect final surface. An excellent score (−4) was also attained for raised and fuzzy grains for both the TH and control samples of Italian alder. However, the TH and control samples showed a level of 6 for pressed grain. A slightly higher frequency of torn grain was recorded for modified samples compared to controls. This was interpreted as an effect of the higher brittleness of TH wood that could result in the generation of torn grain.

Torn grain and pressed grain were recorded on the surface of the control black pine samples with scores of 7 and 5, respectively. Those relatively high values are in line with the practical experiences and confirm the technical problems with processing of untreated pine wood. On the other hand, a noteworthy improvement of the surface quality was observed in the case of TH samples, especially considering the torn grain. Some pressed grain was recorded in both the control and TH samples.

The only sample where fuzzy grain was recorded was the control black poplar wood. This defect disappeared in thermally modified samples, and the only surface imperfection was some pressed grain. Again, the experts’ general assessment highlighted the superior quality of TH pine and poplar surfaces when compared to the control samples.

A total score summing the scores of the single defects was computed in order to give an overview of the machinability of the single species, as shown in Table 3. Deodar cedar was ranked as the best machinable specie both for TH and control samples. Black poplar was ranked as the worst. For all the species, except Italian alder, TH samples were easier to machine compared to untreated samples.

### 3.2. 3D Surface Topography Analysis

**Figure 4:**
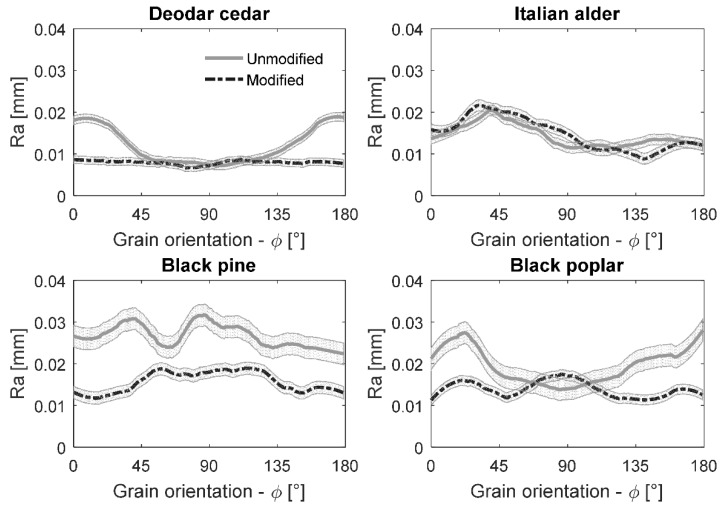
Average roughness (*Ra*) for different grain orientations in thermally modified and control wood samples of diverse species.

A total number of eight half-profiles per specie was averaged and the data was smoothed according to a second-degree polynomial using the Savitzky-Golay algorithm [18] with a span of 20 samples. The scattering around the average value was quantified as the root-mean-square deviation (RMSD). The surface roughness as a function of the grain orientation is shown for all the investigated species in Figure 4. The central thick lines indicate the average *Ra*, while the contours correspond to the scatter of data expressed as RMSD.

The preliminary analysis of the results suggested that, in general, the surface roughness of the control samples resulted in higher roughness values. The only exception was noticed in the case of Italian alder, even if trends in the TH and control samples were very similar. This is in perfect agreement with the visual grading.

The surface roughness varied considerably along the grain orientations, even if the overall trend was different within the evaluated wood species and treatments. The highest roughness was noticed at a grain orientation of 150° < φ < 30° in the case of deodar cedar and black poplar. However, for black pine the worst smoothness was found perpendicular to the grain direction (φ = 90°). The surface roughness was relatively low and constant after machining Italian alder. It was noticed, when combining experts’ visual assessments and objective roughness quantifiers, that *Ra* ≈ 0.02 mm (20 µm) can be considered as a threshold value distinguishing smooth surfaces from rough surfaces. The smoothness perception of the deodar cedar and Italian alder was regularly more favorable than that of other species, which was confirmed by the reduced Ra roughness values. In contrast, very rough black pine and poplar control samples possessed roughness *Ra* > 30 µm.

**Figure 5:**
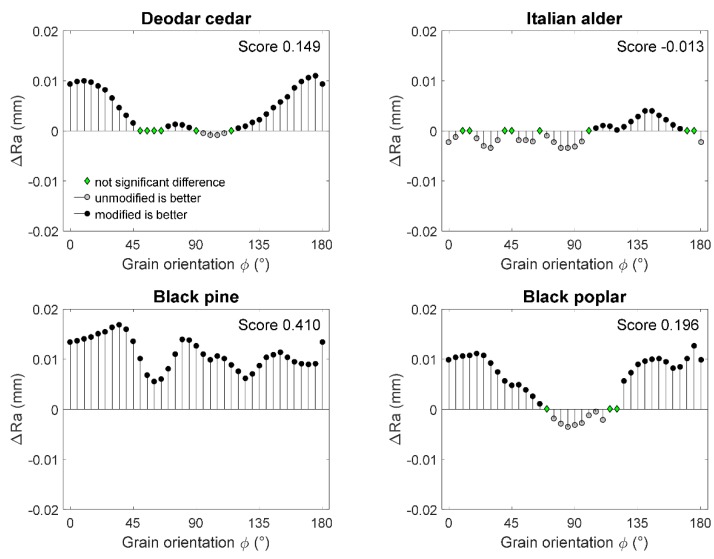
Differences between the average surface roughness for thermally modified and control samples of diverse species.

TH sample data of the eight experimental replicas, on a 5 × 5 degree rotation base, was compared with the control data. The difference was computed and the significance of the difference was tested by a Wilcoxon rank sum test. The results are reported in Figure 5. Where the statistic test has shown no significant difference, Δ*Ra* = 0 is reported (with a green circle), while where significant differences were highlighted, the computed Δ*Ra* is reported. An Δ*Ra* > 0 means that the roughness of the control sample was higher than that of the TH sample. The score is the sum of Δ*Ra* for a single specie and is an overall parameter indicating the average difference between the modified and control samples on the whole range of grain orientations.

Deodar cedar machining TH and control samples resulted in statistically different profiles (score = 0.15). As a general rule, it can be assumed that higher smoothness can be expected on the TH wood surfaces. The positive effect of the modification process is reduced when machining at the end-grain (grain orientation 90°), which indicates a relatively similar surface roughness of treated and control samples. A similar trend was noticed for black poplar, even if control samples appeared to be slightly smoother after cutting in the direction perpendicular to the fibers (Δ*Ra* < 0). The score value of the black poplar was 0.20 and it corresponded to that of deodar cedar.

The surface of the black alder seemed to be smoother when machining control samples against the grain (0° < φ < 90°) as indicated by the statistical test. Inversely, smoother surfaces for TH samples were obtained when machining with the grain (100° < φ < 170°). It has to be stated, however, that the score value was more than 10 times smaller than those of other species, indicating a much smaller statistical difference between the treated and control samples. This tendency was explained with the support of the visual assessment when a slightly higher presence of torn grain was noticed in the modified samples. Also, in this case, the type of defect could be explained with the increased brittleness of the TH wood.

The worst surface roughness was detected on control black pine wood samples. It was statistically demonstrated that thermal modification of this material leads to the dramatic improvement of its machinability. The Δ roughness indicator was constantly >0, signifying reduced values of *Ra* in thermally modified pine wood for all grain orientations.

The values of *Ra* on the whole semi-circumference were summed in order to give a summary index of a single sample. The results are reported in Table 4 and show very good agreement with the results of the visual examination reported in Table 3.

### 3.3. Influence of the Tool Sharpness on the Final Surface Quality

**Figure 6:**
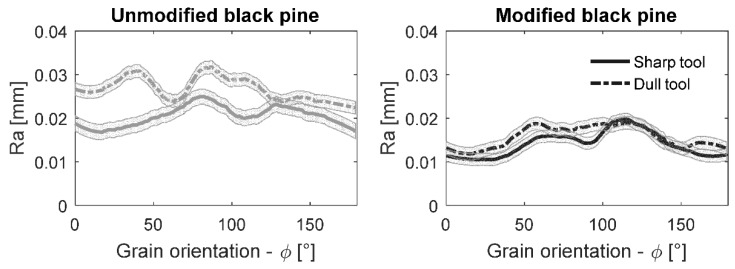
Average roughness for untreated and thermally modified black pine disks at different grain orientations machined with sharp and dull tools.

The continuous progress of the cutting edge wear is a consequence of the tribological action of the tool materials when machining wood (and any other material). There are several studies relating wood surface quality and tool sharpness such as [19,20]. However, it is not reported if the thermal modification of wood affects the surface formation mechanisms while cutting with dull tools. The results obtained within this research are presented in Figure 6 for black pine on the whole range of grain orientations. Black pine was intentionally selected for this test as it has been observed to be the most problematic specie for machining among the four species investigated, both for visual and surface reconstruction grading. The surface roughness appears to be higher when processing wood with a dull tool. It was expected and confirms the state-of-the-art knowledge. However, it was evidenced here that the machining of TH wood results in noticeably smoother surfaces when cutting with either sharp or dull tools. The relation of the smoothness and grain orientation follows the same tendency in the control and treated samples, and it was noticed to be highest for the cutting directions against and perpendicular to the grain (45° < φ < 135°). Such a tendency was more evident in the case of modified pine samples.

**Figure 7:**
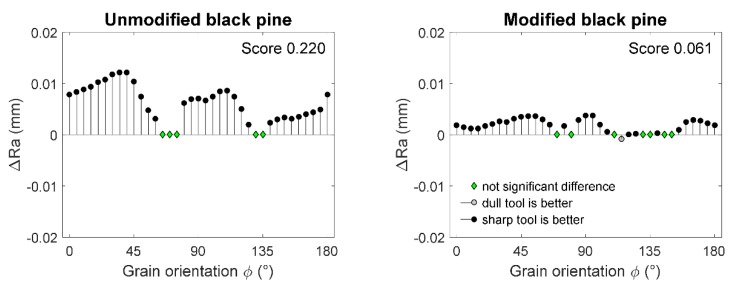
Differences between the average surface roughness after machining thermally modified and unmodified black pine with sharp and dull tools.

The differences and the results of the Wilcoxon rank sum test are shown in Figure 7. It is evident that the excessive dullness of the cutting tool has a negative effect on the generated surface smoothness, as statistically significant Δ*Ra* indicators were of high and positive values for most grain orientations. However, the more noteworthy difference was found for unmodified black pine, as the score coefficient was 400% higher than in modified wood of the same species.

## 4. Discussion

Visual assessment and 3D surface analysis have shown good agreement in terms of the conclusions. The best-quality wood surface was obtained when machining thermally modified samples, especially of species considered problematic for processing. It was particularly evident in the case of black pine or black poplar where the surface roughness was highly reduced due to thermal modification. The positive effect of the material modification was apparent when cutting in difficult conditions (the range of the grain orientations of 135° < φ < 45°). Only Italian alder did not respond to the thermal modifcation with an improved machinability, even if without any modification the wood was already considered easy for processing. It was assumed that the roughness *Ra* ≈ 20 μm is a threshold for considering samples as smooth or rough by means of visual evaluation.

These behaviors can be explained with the different machinability of early and late wood. Early wood is a low-density, high-toughness, and low-stiffness material, while late wood is denser, stiffer and less tough than early wood. For this reason, when machining early wood, large deformations under the effect of the cutting forces are observed and large defects arise. This is especially true when machining against the grain, where early wood often results in fuzzy grain or pressed grain. Late wood, even if it requires higher cutting forces, is stiffer than early wood and generally produces a better surface also if it tends to produce torn grain. The higher tendency of late wood to split, in fact, when machining against the grain, results in the formation of torn grain. The machining of both early and late wood with the grain is less problematic until grain orientations close to the perpendicularity are reached.

Thermal modification, as is well known from the literature [1], has several effects, but from a mechanical point of view we can expect: a reduction of impact toughness, a reduction of the modulus of rupture and a reduction of the work to fracture. For the Young’s modulus, a small increase can be expected for very-low-intensity treatments, and a decrement in all the other cases.

This reaction to the thermal modification treatment has a fundamental role in the increase of the machining quality of early wood which, when it becomes more brittle, is easier to cut without large deformations. This leads to an important increase of the surface quality after machining when species with low-density wood or with a large difference between early and late wood are processed. This is the main reason for the increased quality when machining species such as black pine and black poplar which have a large tendency to form pressed grain or fuzzy grain. For species that are easy to machine, such as deodar cedar and Italian alder, the advantage of machining thermally modified wood is not as high as for the other species. It can also result in small problems such as the increase of the torn grain in Italian alder because of the higher tendency to fracture.

As expected, the use of dull tools resulted in a worse surface quality. However, it was evidenced here that thermal modification has a very positive effect on the generated surface smoothness, by neglecting, to some extent, the consequences of machining with dull tools. This can be attributed to the same reasons discussed previously.

## 5. Conclusions

The machinability of different minor wood species was assessed by visual grading and by three dimensional surface reconstruction. The machining of the sample as well as the three dimensional grading were done according to recently presented original method. A tendency to produce higher smoothness, also with different grain orientations, was observed in Deodar cedar and Italian alder compared to black poplar and black pine that has shown a much lower final surface quality. Both visual and three dimensional reconstruction methods has driven to the same conclusions. The machinability of thermally modified samples was also assessed and compared with the surface quality of control samples. The thermally modified samples have shown to produce a higher smoothness if compared to controls for deodar cedar, black pine and black poplar. The surface improvement is very clear especially where early wood defects are present in the control samples. This was explained with a loss of ductility of the modified material. Modified Italian alder has shown e slightly higher tendency to form torn grain compared to control and resulted in a worse surface rating also if the difference is very small. The final surface quality when machining black pine with a sharp or dull tool were also tested. Control samples have shown a higher smoothness when machined with sharp tool instead than with dull tool. The difference in smoothness between thermally modified wood when machining with dull and sharp tools is much lower. It shows how when machining thermally modified wood, the sharpness of the tool has a lower importance for the final surface smoothness. The three dimensional reconstruction method and the analysis procedure has shown to be an effective and reliable method for the assessment of final surface quality and comparison after machining.

## Figures and Tables

**Table 1:** Wood density of the investigated species before (Control) and after thermal modification (TH). In brackets the standard deviation (SD).

Treatment	Italian Alder	Black Pine	Black Poplar	Deodar Cedar
Control	556 (11)	475 (10)	479 (22)	571 (28)
TH	506 (7)	461 (36)	425 (13)	447 (25)

**Table 2:** Wood moisture content measured by gravimetric method just before the machining process on untreated samples (Control) and thermally modified samples (TH). In brackets the standard deviation (SD).

Treatment	Italian Alder	Black Pine	Black Poplar	Deodar Cedar
Control	11.8 (0.6)	12.6 (0.8)	11.8 (0.7)	12.1 (0.6)
TH	7.6 (0.5)	9.4 (0.9)	7.6 (0.5)	7.1 (0.7)

**Table 3:** Scores of the surface quality of diverse species after machining thermally modified and control samples. The lower the value, the higher the quality (TH—thermal modification).

Specie	Score
Black Pine Control	21.2
Black Pine TH	18.9
Black Poplar Control	20.3
Black Poplar TH	18.4
Deodar Cedar Control	16.3
Deodar Cedar TH	16.0
Italian Alder Control	18.3
Italian Alder TH	19.6

**Table 4:** Scores of the surface quality of diverse species after machining thermally modified and control samples. The lower the value, the higher the quality (TH—thermal modification).

Specie	Score (mm)
Black Pine Control	9.586
Black Pine TH	5.301
Black Poplar Control	7.012
Black Poplar TH	5.154
Deodar Cedar Control	4.279
Deodar Cedar TH	2.849
Italian Alder Control	5.009
Italian Alder TH	5.640
